# Body mass index and the risk of rheumatoid arthritis: a systematic review and dose-response meta-analysis

**DOI:** 10.1186/s13075-015-0601-x

**Published:** 2015-03-29

**Authors:** Baodong Qin, Min Yang, Haitao Fu, Ning Ma, Tingting Wei, Qingqin Tang, Zhide Hu, Yan Liang, Zaixing Yang, Renqian Zhong

**Affiliations:** Department of Laboratory Diagnostics, Changzheng Hospital, Second Military Medical University, 415 Fengyang Road, Shanghai, 200003 China; Department of Laboratory Diagnostics, Sir Run Run Shaw Hospital, Zhejiang University School of Medicine, Hangzhou, Zhejiang 310000 China

## Abstract

**Introduction:**

The evidence from published studies on the association between obesity and rheumatoid arthritis has been contradictory. To clarify the association between obesity and rheumatoid arthritis, we conducted a systematic review and dose-response meta-analysis to assess the relationship between body mass index and rheumatoid arthritis risk.

**Methods:**

A systematic literature search of PubMed and Embase (up to 12 July 2014) was performed to identify all eligible published reports. The pooled relative risk results with corresponding 95% confidence intervals of rheumatoid arthritis development were estimated using a random-effects model.

**Results:**

Eleven eligible related citations fulfilled the inclusion criteria and were included in the study. Compared with individuals with a body mass index under 30, obese individuals showed an association with a significantly increased risk of rheumatoid arthritis (relative risk = 1.25, 95% confidence interval: 1.07 to 1.45, *P*_heterogeneity_ <0.01, I^2^ = 63%). Compared to normal weight subjects, the pooled relative risks for rheumatoid arthritis were 1.31 (1.12 to 1.53) and 1.15 (1.03 to 1.29) for the categories of obese and overweight, respectively. In the dose-response analysis, there was evidence of a nonlinear association (*P*_nonlinear_ = 0.005) and the estimated summary relative risk for a 5-unit increment was 1.03 (95% confidence interval: 1.01 to 1.05, *P*_heterogeneity_ = 0.001, I^2^ = 70.0%).

**Conclusions:**

An increase in body mass index can contribute to a higher risk for rheumatoid arthritis development. However, the finding also highlights the need for research on the association between body mass index and rheumatoid arthritis risk with adjustment for more confounding factors.

**Electronic supplementary material:**

The online version of this article (doi:10.1186/s13075-015-0601-x) contains supplementary material, which is available to authorized users.

## Introduction

Rheumatoid arthritis (RA) is the most common autoimmune disease, affecting approximately 0.5 to 1% of the adult population worldwide, and it is characterized by diffuse synovial inflammation and destruction [1-3]. It primarily affects women, with a female to male ratio from about 2:1 to 3:1 [1]. Although the etiology and pathogenic mechanism underlying the development of RA remain unclear, the combination of a susceptible genetic background interplaying with environmental factors has been considered to be associated with the development of this complex disorder [4]. It has been reported that there are several risk factors contributing to the initiation and promotion of this complex disorder, such as age, gender, hormonal levels, alcohol, cigarette smoking, socioeconomic status, and dietary habits [5-8].

Obesity is a major health issue affecting many people. For example, about two thirds of adults and one third of children in the USA are obese [9]. Obesity has contributed to increased morbidity and mortality, and people who are overweight or obese are at increased risk of several diseases. Although body mass index (BMI) cannot measure the percentage of body fat and accurately reflect obesity, it is also considered to be a useful indicator for obesity. In a previous study, the association of obesity with RA was demonstrated [10], but another study examining the association of BMI with RA risk yielded different and controversial results [11]. Several prior studies have observed a positive relationship between BMI and increased risk of developing RA, suggesting obesity is an important and modifiable risk factor for RA [10,12]. Conversely, some studies did not identify this association [11,13,14].

Due to these conflicting results, we conducted a systematic review and meta-analysis of the available literature to quantitatively assess the effect of BMI on RA development. In addition, the exact shape of the dose-response relationship between BMI and RA risk has not been clearly defined; therefore the other aim of this study was to clarify the dose-response relationship between BMI and RA risk.

## Materials and methods

### Research strategy

The systematic review and meta-analysis was conducted based on the guidelines set by the Meta-analysis of Observational Studies in Epidemiology (MOOSE) [15]. For selection of eligible studies, two authors (BDQ and MY) independently performed a literature search in PubMed and Embase databases (up to 12 July 2014). In PubMed, the comprehensive search strategies included the Mesh terms and keywords [(“Obesity”[Mesh] or “Obesity” or “Obese” or “Body Mass Index”[Mesh] or “Body Mass Index” or “Overweight”[Mesh] or “Overweight”) and (“Arthritis, Rheumatoid”[Mesh] or “Rheumatoid Arthritis”)]. The search strategy used in Embase included (exp “Rheumatoid Arthritis” or “Rheumatoid Arthritis”) and (exp “Obesity” or “Obesity” or exp “Body Mass Index” or “Body Mass Index” or Exp “Overweight” or “Overweight”). The search was limited to the English language, and no other restrictions were imposed on ethnicity, human subjects or geographic regions. The references of relevant articles were also reviewed for further analysis.

### Study selection

The studies were limited to relevant articles concerning the association of BMI and RA risk: (a) the study had to have a case-control or cohort design; (b) the study reported risk estimates with the corresponding 95% confidence intervals (95% CI), or sufficient data for extraction or assessment; and (c) obesity, being overweight, and BMI were the exposures of interest. For a dose-response meta-analysis, the study must have given risk estimates at three or more quantitative categorized levels. Studies that did not report sufficient published data or original data were excluded. When studies on the same or overlapping populations were published, only the one with the most detailed information or the largest sample size was eligible. Reviews, case reports, mechanism studies, and unpublished studies, as well as non-human studies were excluded.

### Data extraction and assessment of study quality

The primary variables of interest were the BMI categories and RA risk estimates including relative risks (RRs) and/or odds ratios (ORs) with their 95% CIs. We preferred using the RRs and/or ORs from the maximally adjusted model to reduce the possibility of potential residual confounding. Also, a standard reporting form was established according to the information extracted from each study, including the first author’s name, country or region, publication year, age of participants, diagnosis criteria for RA, evaluation method of BMI, BMI categories, sample size, and the number of patients in case or control groups in each BMI category. The Newcastle-Ottawa scale (NOS) scoring system was used to assess the methodological quality of the included studies. The NOS included eight items that were categorized into three dimensions (selection, comparability, and exposure). The scoring system provides a summary numeric score of quality ranging from 0 to 9 stars [16]. The final results were compared by two authors (BDQ and MY) and any disagreements were resolved by consensus with a third party (ZXY or RQZ).

### Statistical analysis

The mean or median level of BMI for each category was considered with the corresponding RR and/or OR. When the mean or median of the BMI category was not provided, the midpoint of the upper and lower boundaries in each BMI category was assigned as the average level. When the highest or lowest categories were open-ended, we assumed that the open-ended interval length was the same as the adjacent interval. The BMI (kg/m^2^) was classified into three categories: obesity, (>30.00); overweight (25.00 to 29.99); and normal weight (18.50 to 24.99) [17]. The pooled RR with its 95% CI was calculated in a random-effects model. The subgroup meta-analysis was also performed to estimate the specific RR. The dose-response meta-analysis was conducted using the method described by Greenland and Longnecker [18]. We also computed study-specific linear trends and 95% Cls from the natural logs of the RRs and Cls across categories for BMI. In the method, the distribution of cases and non-cases, or person-years and the ORs and/or RRs with the variance estimate for at least three quantitative BMI categories, were required to be known. The dose-response relationship was presented in a forest plot with a 5 kg/m^2^ BMI increment. A restricted cubic splines model, with knots fixed at percentiles 10%, 50%, and 90% through the distribution, was estimated with least-square regression, taking into account the correlation within each set of published RR and/or ORs. Then these risk estimates were combined in a multivariate random-effects meta-analysis using the restricted maximum likelihood method [19].

The *χ*^2^-based Q test and I^2^ statistic were used to judge heterogeneity in the studies. For the Q test, *P* <0.10 was indicated to be representative of statistically significant heterogeneity, and the I^2^ statistic represented the percentage of total variation contributed by a between-study variation ranging from 0 to 100% [20]. A subgroup analysis by study design, gender, and region was also carried out to examine the effects of these factors on heterogeneity. In addition, meta-regression was also conducted to find the potential sources of heterogeneity. The publication bias was assessed using funnel plots, Egger’s test, and Begg’s test. Sensitivity analysis was conducted to determine the degree that each single study affected the overall ORs, and to evaluate whether the results were robust, using the one-study remove approach. All statistical analyses were done using STATA 12.0 software (Stata Corp, College Station, TX, USA). *P* values less than 0.05 were considered significant.

## Results

### Study selection

**Figure 1:**
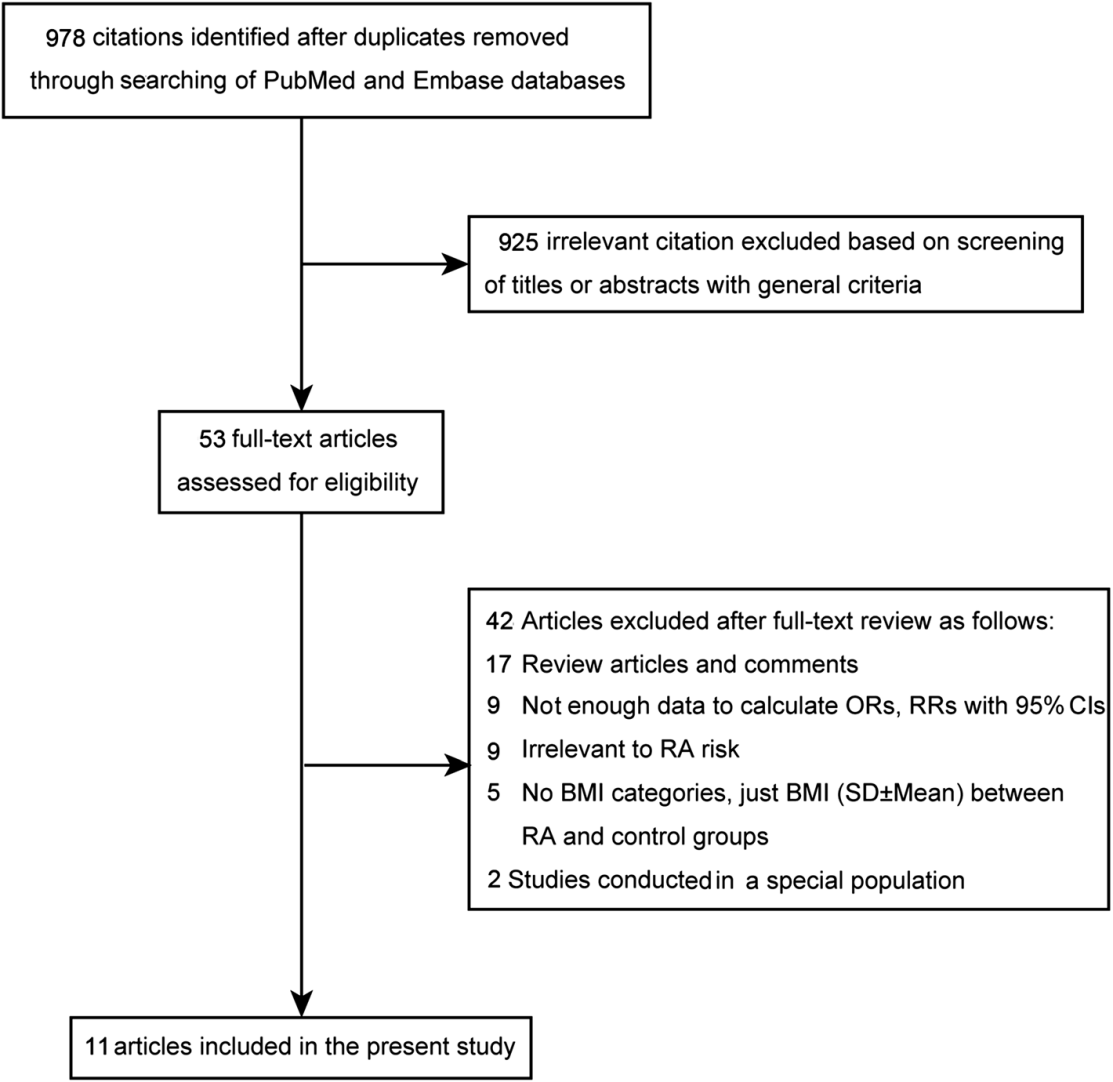
**Flowchart of article identification, inclusion, and exclusion.** BMI: body mass index; CI: confidence interval; OR: odds ratio; RA: rheumatoid arthritis; RR: relative risk.

The derivation of the relevant studies included in the present meta-analysis is shown in Figure 1. A total of 978 non-overlapping publications were identified from the previously described databases. After screening of abstracts or titles, 925 irrelevant studies were excluded, leaving 53 eligible studies for the full-text review and evaluation eligibility. After detailed assessment, 11 studies that met the inclusion criteria were left for final inclusion [11-14,21-27]. No other new eligible study was identified from the manual research of reference lists of those 11 articles. The agreement between reviewers for the eligibility of studies was 100%.

### Characteristics of included studies

The main characteristics of each study are described in Table 1. All 11 eligible articles were published in English between 1994 and 2014. Four studies were conducted in American populations [11,21,24,27] and seven in European populations [12-14,22,23,25,26]. Seven studies were designed as case-control studies [12-14,21-24] and four studies were cohort studies [11,25-27]. The method of case ascertainment in most studies was based on the American College of Rheumatology (ACR) 1987 criteria [28]. Four studies were conducted in the female population [11,21,26,27]. One study only discussed the role of obesity in the development of RA without BMI categories [24]. Two studies did not yield the number of RA cases and person-years [25,26].

**Table 1:** **Summary of studies included in the present study**

**Author**	**Year**	**Country**	**Study design**	**Gender (RA/control)**	**Age (years)**	**Assessment of BMI**	**Sample size (RA/control)**	**Diagnosis criteria**	**Rates of obesity (RA/control)**	**BMI categories**	**Adjustment for covariates**
Voigt *et al*. [21]	1994	USA	Case-Control Study	Female	18-64	Self-reported	349/1,456	Diagnostic Criteria 1958^e^	29.5%/24.8%	12.94-20.43,	Age, smoking status
				Female						20.44-22.51,	
										22.52-25.82,	
										25.83-52.86	
Symmons *et al*. [22]	1997	UK	Case-Control Study	Mixed	18-70	Self-reported	90/93	ACR 1987^f^	17.6%/7.7%	<20, 20-24.9, 25-29.9, >30	Smoking status, social class
				Mixed							
Uhlig *et al*. [12]	1999	Norway	Case-Control Study	Mixed	20-79	Self-reported	347/5,725	ACR 1987	7.8%/4.6%	<25, 25-29.9, >30	Age, sex, marital status, employment category, formal education, current smoking status
				Mixed							
Pedersen *et al*. [23]	2006	Denmark	Case-Control Study	Mixed^b^	18-65	Self-reported	505/752	ACR 1987	9.3%/6.8%	<18.5, 18.5-25, 25-30, >30	Birth year, year of RA diagnosis, gender
				Mixed							
Rodriguez *et al*. [13]	2009	UK	Case-Control Study	Mixed	20-79	Medical Examination	559/4,234	NA	12.1%/12.5%	<20, 20-24.9, 25-30, >30	Age, gender, referrals, smoking status, alcohol consumption, pregnancy status
				Mixed							
Wesley *et al*. [14]	2013	Sweden	Case-Control Study	Mixed Mixed	18-70	Self-reported	2,748/3,444	ACR 1987	13.7%/13.0%	<25, 25-30, >30	Smoking status, alcohol consumption, education level, menopausal status, physical activity, parity, fatty fish consumption
Crowson *et al*. [24]	2013	USA	Case-Control Study	Mixed	55.9 ± 15.7	Medical Examination	813/813	ACR 1987	40.3%/35.7%	<30, >30	Age, gender, smoking status
				Mixed							
Cerhan *et al*. [11]	2002	USA	Cohort Study	Female Female	55-69	Self-reported	31,336^c^	ACR 1987	25.9%^d^	<23.4, 23.4-25.8, 25.9-29.2, >29.2	Age
Lu *et al*.^a^ [27]	2014	USA	Cohort Study	Female	25-55	Medical Examination	218,623	ACR 1987	27.8%^d^	18.5-24.9, 25-29.9, >30	Age, community median income, smoking status, alcohol consumption, physical activity, parity, breastfeeding status, postmenopausal use Postmenopausal Hormone use.
				Female							
Harpsoe *et al*. [26]	2014	Denmark	Cohort Study	Female	27.4- 33.3	Self-reported	75,088	ICD Code	-	<18.5,18.5-25, 25-30, >30	Smoking status, alcohol consumption, parity, socio-occupational status
				Female							
Lahiri *et al*. [25]	2014	UK	Cohort Study	Mixed Mixed	40-79	Self-reported	25,271	ACR1987	-	<25, 25-30, >30	Age, gender, smoking status, breastfeeding status, alcohol consumption

^a^Two cohorts (NHS1 and NHS2) was included in this study. ^b^The studies reported the association of BMI and RA risk in both female and male population. ^c^The total sample size in the cohort studies. ^d^The prevalence rate of obesity in RA patients. The prevalence rate of obesity in non-RA individuals were not available. ^e^Diagnostic Criteria is a revised version of criteria for rheumatoid arthritis published in 1958 [42]. ^f^ACR1987 is the American College of Rheumatology criteria for rheumatoid arthritis published in 1987 [28]. BMI: body mass index; ICD: International Classification of Diseases [43]; NA: No stated; RA: rheumatoid arthritis.

### Study quality

According to the NOS score system, the quality rating of individual studies ranged from five to eight stars on a scale of nine, including seven studies scoring eight stars, three studies scoring seven stars, and one study scoring five stars (Additional file 1).

### Body mass index and rheumatoid arthritis risk

**Figure 2:**
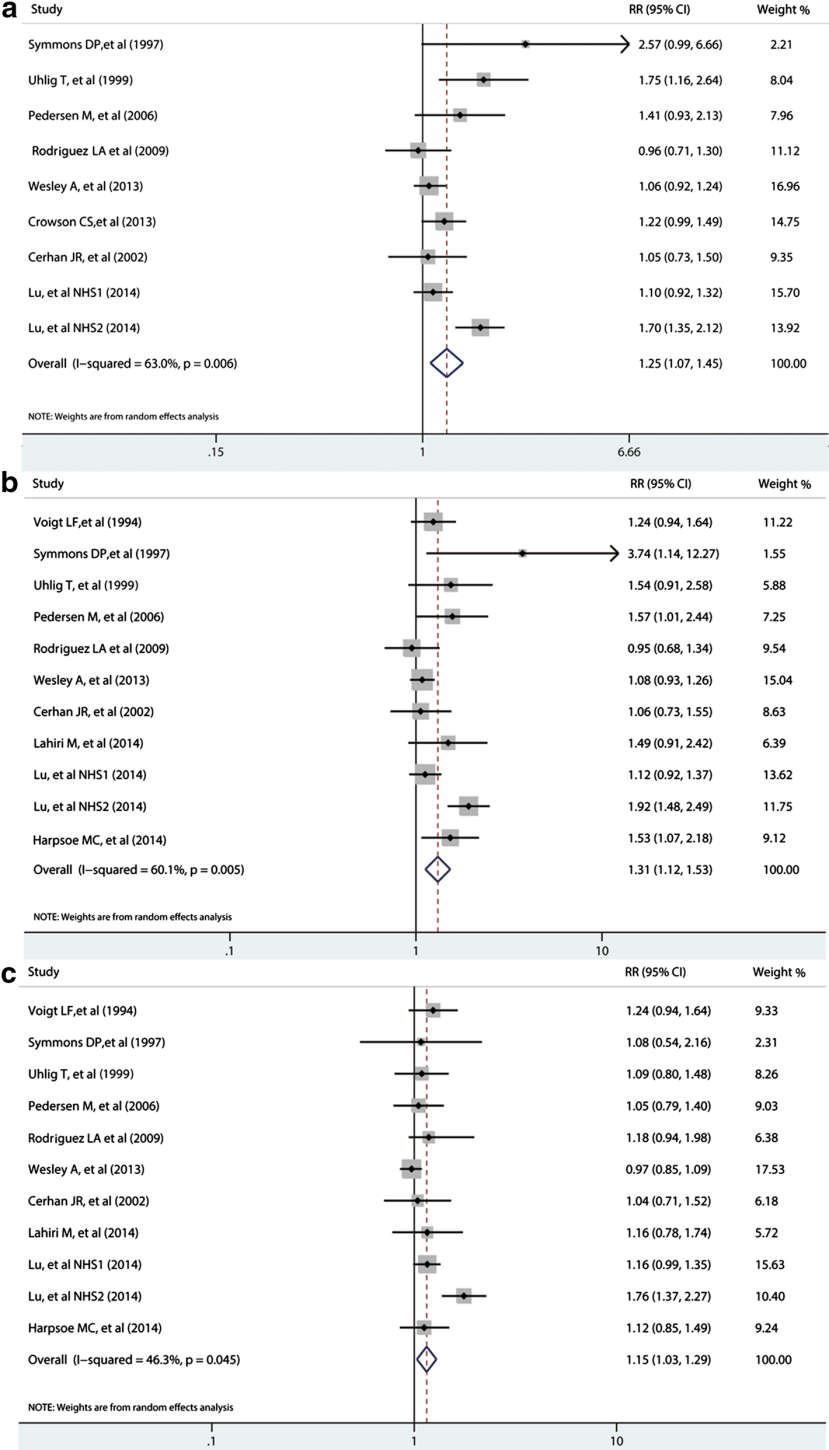
**Summary of relative risks of rheumatoid arthritis. (a)** obesity versus non-obesity; **(b)** obesity versus normal weight; **(c)** overweight versus normal weight; meta-analysis using a random-effects model. CI: confidence interval; RR: relative risk.

Compared to individuals with a BMI score under 30, obese individuals had a significantly increased risk of RA (RR = 1.25, 95% CI: 1.07 to 1.45), but with high heterogeneity (I^2^ = 63%, *P* <0.01 for Q statistic; Figure 2a). Compared to the reference category of normal weight, the pooled RRs of RA were 1.31 (1.12 to 1.53; Figure 2b) and 1.15 (1.03 to 1.29; Figure 2c) for the categories of obese and overweight, respectively. Some evidence of significant heterogeneity was also observed across these studies (I^2^ = 60.1%, *P* <0.01; I^2^ = 46.3%, *P* = 0.045). Because Voigt *et al*. yielded the relative risks for BMI >25 rather than obesity or being overweight, the overall RRs of RA risk were 1.33 (1.11 to 1.58) and 1.15 (1.03 to 1.29) for obesity and being overweight, when excluding Voigt *et al*. [21], respectively.

### Subgroup analysis

**Figure 3:**
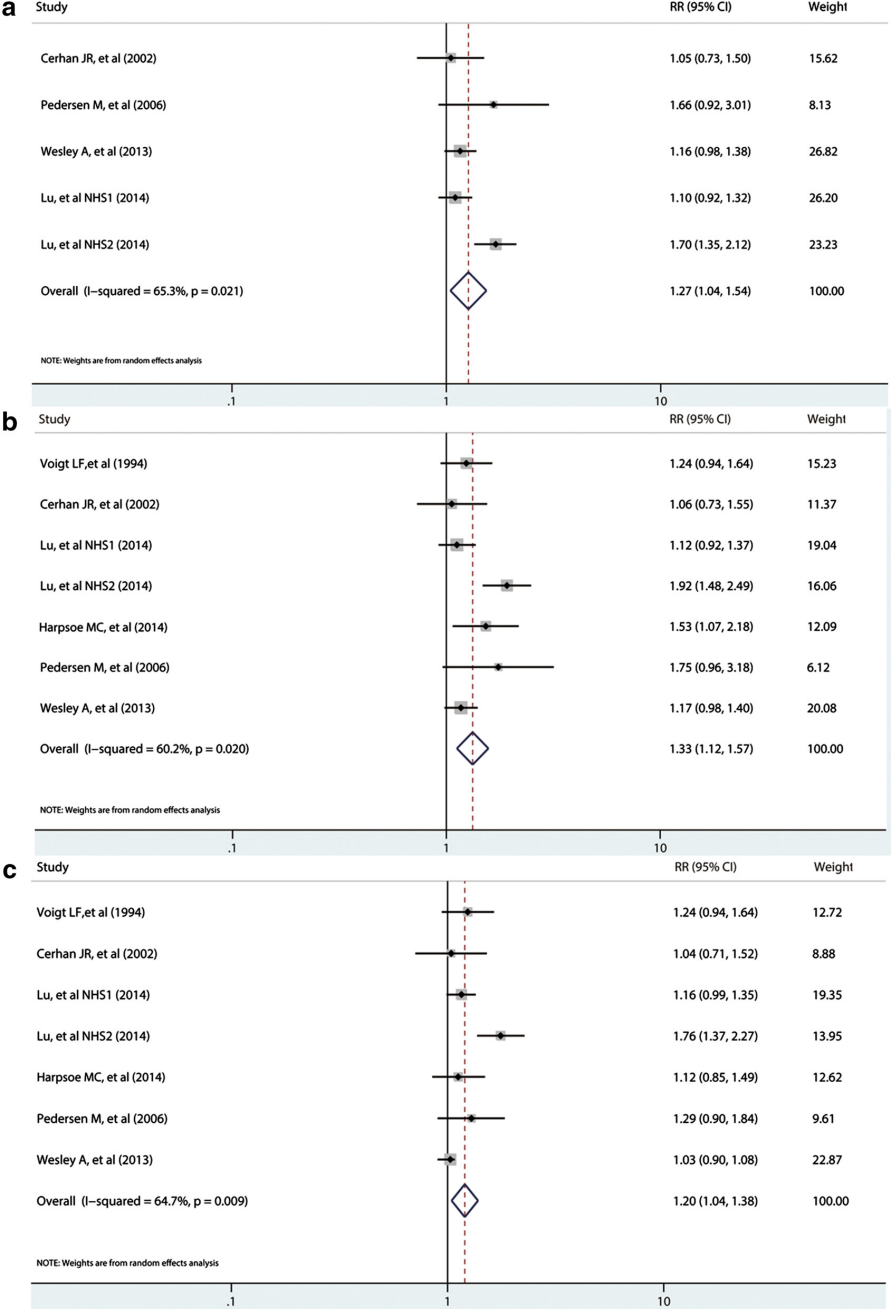
**Summary of relative risks of rheumatoid arthritis in female populations. (a)** obesity versus non-obesity; **(b)** obesity versus normal weight; **(c)** overweight versus normal weight; meta-analysis using a random-effects model. CI: confidence interval; RR: relative risk.

Of the included studies, five studies containing six cohorts assessed the role of BMI in the development of RA in female populations. The subgroup analysis in females revealed that the RRs of RA between obesity, overweight, and normal weight were 1.33 (1.12 to 1.57; Figure 3b) and 1.20 (1.04 to 1.38; Figure 3c), respectively. Significantly, heterogeneity was also found (I^2^ = 60.2%, *P* = 0.02; I^2^ = 64.7%, *P* <0.01). The comparison of female obesity with female non-obesity showed that obese individuals had a 27% significantly increased risk for developing RA (RR = 1.27, 95% CI: 1.04 to 1.54) with great heterogeneity (I^2^ = 65.3%, *P* = 0.02; Figure 3a).

Specific data for the association between BMI and RA risk were also stratified on the basis of study design, including case-control studies and cohort studies. Four cohort studies reported the influence of BMI on RA development in a defined geographical area containing at least 25,271 participants. However, the number of subjects in eight case-control studies concerning this relationship was relatively small, ranging from 90 to 2,748 RA cases. Subgroup meta-analysis by study design showed that the pooled RRs for obesity versus non-obesity, obesity versus normal weight, and overweight versus normal weight were 1.22 (1.01 to 1.46), 1.22 (1.01 to 1.48), and 1.03 (0.94 to 1.14) across case-control studies, while they were 1.27 (0.92 to 1.73), 1.39 (1.08 to 1.78), and 1.24 (1.03 to 1.50) for cohort studies (Additional file 2).

All included studies were conducted in Europe (n = 7) and North America (n = 4). The subgroup meta-analysis stratified by region showed that the RRs were 1.26 (0.98 to 1.62), 1.31 (1.07 to 1.61), and 1.03 (0.94 to 1.13) for obesity versus non-obesity, obesity versus normal weight, and overweight versus normal weight in European populations, while they were 1.26 (1.02 to 1.55), 1.31 (1.00 to 1.71), and 1.29 (1.04 to 1.60) in North American populations (Additional file 3). After stratification, great heterogeneity could also be found across those studies in different regions.

Among all eleven included studies, three studies with four cohorts investigated the association of BMI on RA patients with and/or without antibodies to citrullinated peptide antigens (ACPA) seropositivity [14,23,27]. The subgroup analysis stratified by ACPA seropositivity revealed that RRs were 1.34 (0.98 to 1.82), 1.47 (0.94 to 2.30), and 1.18 (0.75 to 1.87) for obesity versus non-obesity, obesity versus normal weight, and overweight versus normal weight in ACPA-seronegative RA patients, and 1.39 (1.07 to 1.79), 1.47 (1.09 to 1.98), and 1.34 (1.08 to 1.65) in ACPA-seropositive RA patients, respectively.

### Dose-response analysis

**Figure 4:**
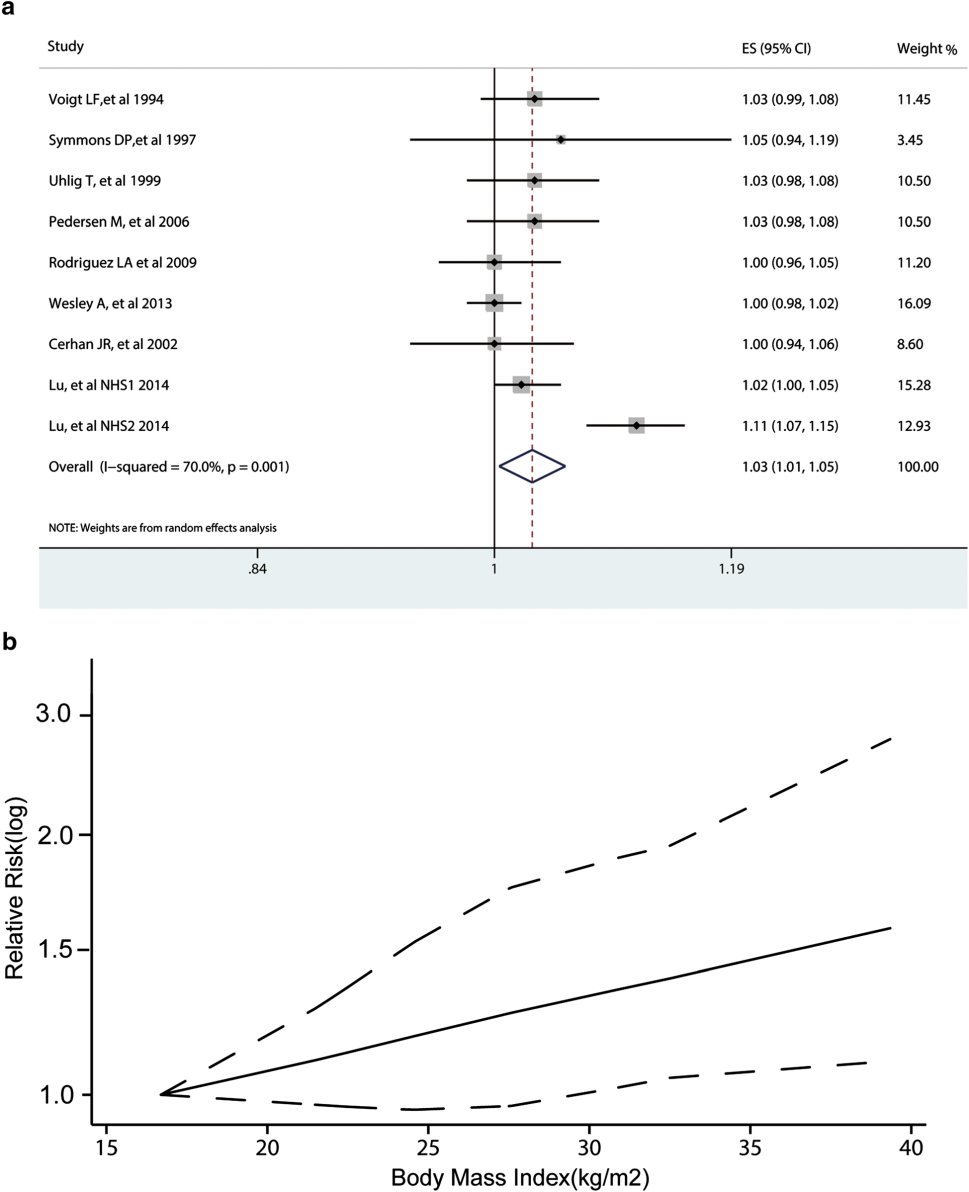
**Dose-response meta-analysis between body mass index and rheumatoid arthritis risk. (a)** RR of RA risk per 5 kg/m^2^ increase in BMI; **(b)** nonlinear dose-response association, BMI was modeled with a nonlinear trend (black continuous line) in a random-effects meta-regression model. Long-dashed black lines represent 95% confidence intervals. Short-dashed black lines represent the linear trend. The vertical axes are on a log scale. BMI: body mass index; RA: rheumatoid arthritis; RR: relative risk. ES: Effect Size.

Eight studies including nine cohorts reported RRs for categorized BMI levels. A dose-response analysis including these studies was conducted. The summary RR of RA risk per 5 kg/m^2^ increase in BMI was 1.03 (1.01 to 1.05; Figure 4a). The subgroup analysis by region showed that the RR of RA risk per kg/m^2^ increase in BMI was 1.04 (0.99 to 1.09) and 1.01 (0.99 to 1.03) in North American and European populations (Additional file 4), respectively, with significantly greater heterogeneity across North American studies (I^2^ = 82.1%, *P* <0.01) than European studies (I^2^ = 0.0%, *P* = 0.609). The subgroup meta-analysis by study design revealed that the RRs were 1.01 (0.99 to 1.03) for case-control studies and 1.04 (0.98 to 1.11) for cohort studies (Additional file 4). The RR of RA risk per 5 kg/m^2^ increase in BMI was 1.04 (0.99 to 1.09) in female populations (Additional file 4). Meta-regression analysis showed that the potential source of noticeable heterogeneity for dose-response analysis may not be attributed to region (*P* = 0.307), gender (*P* = 0.307), sample size (*P* = 0.137), study design (*P* = 0.304), and publication year (*P* = 0.762). In addition, there was evidence of a nonlinear association between BMI and RA risk (*P*_non-linearity_ = 0.005; Figure 4b).

### Publication bias

The funnel plot for the pooled RRs of RA risk per 5 kg/m^2^ increase in BMI did not show publication bias visually (Additional file 5), and the *P* values for Begg’s test and Egger’s test were 0.532 and 0.915, respectively. The assessment of bias also showed that there was no publication bias for obesity versus non-obesity (*P*_Begg’s test_ = 0.283, *P*_Egger’s test_ = 0.992), obesity versus normal weight (*P*_Begg’s test_ = 0.119, *P*_Egger’s test_ = 0.819), and overweight versus normal weight (*P*_Begg’s test_ = 0.640, *P*_Egger’s test_ = 0.975). The funnel plots are shown in Additional file 5. In addition, no significant publication bias was detected in the other meta-analyses.

### Sensitivity analysis

The pooled RRs for obesity versus non-obesity ranged from 1.24 (1.07 to 1.44) to 1.35 (1.14 to 1.59), after excluding one study at a time in the sensitivity analysis. Obesity versus normal weight ranged from 1.21 (1.07 to 1.36) to 1.36 (1.14 to 1.61), and overweight versus normal weight ranged from 1.07 (0.99 to 1.16) to 1.20 (1.08 to 1.33). Excluding Voigt *et al*., the relative risk of RA development for obesity and being overweight was 1.31 (1.08 to 1.60) and 1.15 (1.02 to 1.29) compared with normal weight, respectively [21] (Additional file 6). The summary RR of RA risk per 5 kg/m^2^ increase in BMI did not significantly change after one study at a time was removed; for example, it was 1.01 (1.00 to 1.03; *P* = 0.058) after omitting the NHS2 cohort study of Lu *et al*., and 1.03 (1.01 to 1.06) after omitting the study of Wesley *et al*. [14,27].

## Discussion

With the performance of genome-wide association studies, there have been major advances in our understanding of genetic risk [29]. In addition, there has also been renewed interest in environmental factors, especially lifestyle factors such as smoking, breast feeding, and alcohol intake, which have been identified to have a dose-related association with diseases. As a potential risk factor, several studies have investigated the association of obesity with RA development; however, the relationship remains poorly understood. We therefore conducted a systematic review to comprehensively summarize the current literature on the association of BMI with RA development. We also performed a dose-response meta-analysis to assess whether there was a linear or nonlinear dose-response relationship between BMI and RA risk.

Eleven studies regarding the association between BMI with RA risk showed a positive association between obesity and RA. Compared with non-obese individuals, individuals who were obese had a 24% increased risk for RA development. Individuals who were obese or overweight had a 31% and 15% increased risk for RA in comparison to individuals of normal weight, respectively. The results obtained from a subgroup meta-analysis showed that the RR for RA was high in females compared to mixed populations. One explanation for this phenomenon may be that female sex hormones could modify the effect of obesity on RA risk [30,31]. The pooled RRs across studies in North American populations were also relatively higher than those of European populations. This may due to most North American studies being designed as cohort studies, which may give relatively higher RRs. For example, the summary RRs from case-control studies were lower than those of cohort studies for obesity versus normal weight (1.22 versus 1.39) and overweight versus normal weight (1.03 versus 1.24), while the RR for obesity versus non-obesity was higher across case-control studies (1.32 versus 1.27). Besides, the results from a sensitivity analysis revealed that the effect of obesity on RA risk in the NHS2 cohort study of Lu *et al*. was much larger than that observed in the other studies. Lu *et al*. found that there was not a significant association between BMI and RA when restricting to RA cases diagnosed after 55 years of age, and 83% of RA cases in that study were diagnosed at or before 55 years of age [27]. Hence, the potential explanation for higher risk of BMI on RA risk in the NHS2 study by Lu *et al*. may be that the age of NHS2 participants was relatively younger.

In addition, all included studies were conducted in North American and European populations. Data for other continents were lacking, and investigators in these regions should pay more attention to the assessment of BMI and RA. Because of the different lifestyles, different living environments, and different economic development between developed countries and developing countries, obesity or increased BMIs may have different effects on the occurrence of disease around the world, especially in Africa. In two cross-sectional studies from African countries, the investigator found that the traditional factors, such as obesity, were not independent risk factors for cardiovascular disease in RA patients when comparing developing black African populations and developed Caucasian populations [32,33]. These findings suggested that obesity or an increased BMI may have different associations with disease in developing regions or developed regions. In a study by Dessein *et al*., the authors found BMI, waist circumference, and hip circumference to be relatively lower in African RA individuals than non-RA individuals, and the incidence of Africans with RA decreased overall with abdominal adiposity [34]. These results further support the hypothesis that obesity or BMI may have a diverse influence on RA risk in different regions.

RA can be divided into two major subsets based on the presence of ACPA. Recent genome-wide association studies have shown that significant risk allele frequencies were different between ACPA-positive and ACPA-negative RA patients, which showed a difference in distinct genetic etiologies of those two RA subsets, and provided further support for the need to consider them separately. The association of BMI with disease risk has also been identified to be different in those two subsets [35]. In the study by Wesley *et al*., findings indicated that obesity is related to ACPA-negative RA development in women, and showed an inverse association between BMI and ACPA-positive RA in men [14]. In the study by Pedersen *et al*., BMI was also found to be strongly and selectively associated with ACPA-negative RA [23]. However, the study by Lu *et al*., which contained two cohorts, concluded that the RR of RA was elevated among overweight and obese women both in ACPA-positive and ACPA-negative RA [27]. The results from subgroup analysis by ACPA seropositivity revealed the association of BMI or obesity with RA risk in ACPA-positive RA rather than ACPA-negative RA, suggesting that BMI plays a different role in these two major RA subsets based on the presence of ACPA. However, the great heterogeneity may reflect modification of the relationship of BMI with ACPA-seropositive or ACPA-seronegative RA, and other confounding factors, such as age and gender need to be studied.

Although the mechanism by which obesity or higher BMI could lead to RA remains unclear, there are several potential mechanisms. First is the association between obesity and inflammation. Obesity is often considered a systemic inflammatory condition with increased levels of inflammatory cytokines, including tumor necrosis factor-alpha and interleukin-6 [36]. These inflammatory cytokines could promote the inflammatory response of individuals. Leptin, as a pro-inflammatory adipokine, could be secreted excessively in obesity by adipocytes. Previous studies have identified leptin as a potent immune modulator, which could sustain autoreactive cell proliferation and impact inflammation [37]. Both leptin and inflammatory cytokines are implicated in the development of autoimmune diseases. Second, altered sex hormones metabolism in obese subjects may share a similar mechanism in the etiology of RA. Obese individuals have higher levels of estrogens and androgens [38,39]. Sex hormones have also been shown to play a role in the development of RA, which could be modified by obesity [40]. Besides, the link between obesity and autoimmune diseases could be driven by a genetic variation which could predispose individuals to both conditions [41].

Of note, there were several limitations in the present study. First, the number of included studies was relatively small. Some of the eligible studies were case-control studies, which were more prone to bias. For example, case-control studies were at a major risk of recall bias, and participants in several studies were asked to report their BMI before interview. The assessment of BMI in subjects from seven included studies was obtained by a self-reported questionnaire, which may influence the accuracy of the data. Second, due to the lack of data provided by each study on the association of BMI categories and RA risk, secondary calculations were required in some cases. These secondary calculations may not exclude the influence of some bias factors on the results. Third, eligible studies only consisted of published data; unpublished data were not identified. This suggests that publication bias cannot be absolutely excluded even though no significant publication bias was detected. Because the high heterogeneity was still observed in subgroup meta-analysis by gender, region, ACPA, and study design, other factors may be the cause of this great heterogeneity, such as rheumatoid factor. However, the role of BMI in RA patients with or without rheumatoid factor could not be determined due to insufficient data. Therefore, it was impossible to completely exclude the influence of inherent confounding factors. In addition, another major limitation is the differences in adjustment for covariates. As shown in Table 1, different covariates were used for multivariable analysis among all included studies. The meta-analysis of included studies inherits the limitation of the original studies. Although most included studies adjusted for potential confounders such as age, gender, smoking, alcohol, and parity, the possibility of residual confounding cannot be ruled out in these studies.

## Conclusions

This study evaluated the association of BMI with RA risk by systematically reviewing the relevant literature. The results suggested that an increase in BMI could contribute to higher risk for RA. However, future research should employ prospective studies with adjustment for more confounding factors.

## Additional files

Additional file 1:
**Methodological quality of case-control studies/cohort studies according to the Newcastle-Ottawa quality assessment scale.**
Additional file 2:
**Overall RR of rheumatoid arthritis stratified by study design. (a)** obesity versus non-obesity; **(b)** obesity versus normal weight; **(c)** overweight versus normal weight; meta-analyses using a random-effects model. CI: confidence interval; RR: relative risk. Additional file 3:
**Overall RR of rheumatoid arthritis stratified by region. (a)** obesity versus non-obesity; **(b)** obesity versus normal weight; **(c)** overweight versus normal weight; meta-analyses using a random-effects model. CI: confidence interval; RR: relative risk. Additional file 4:
**RRs of rheumatoid arthritis risk per 5 kg/m2 increase in body mass index stratified by region, study design, and gender. (a)** region; **(b)** study design; **(c)** gender; meta-analyses using a random-effects model. CI: confidence interval; RR: relative risk. Additional file 5:
**Funnel plots for obesity versus non-obesity, obesity versus normal weight, overweight versus normal weight, and dose-response per 5 kg/m2 increase in body mass index.** RR: relative risk. Additional file 6:
**Sensitivity analyses for obesity versus non-obesity, obesity versus normal weight, overweight versus normal weight, and dose-response by per 5 kg/m2 increase in body mass index.**

## Abbreviations

ACPA: Antibodies to citrullinated peptide antigens
BMI: Body mass index
CI: Confidence interval
OR: Odds ratio
RA: Rheumatoid arthritis
RF: Rheumatoid factor
RR: Risk ratio
