# Supplementary material for: Body mass index and the risk of rheumatoid arthritis: a systematic review and dose-response meta-analysis
Source: Arthritis Res Ther. 2015 Mar 29;17(1):86. doi: 10.1186/s13075-015-0601-x (PMC4422605; doi:10.1186/s13075-015-0601-x)
Supplement: Additional file 1: — Methodological quality of case-control studies/cohort studies according to the Newcastle-Ottawa quality assessment scale. [file 13075_2015_601_MOESM1_ESM.doc]

Supplementary. Table: Methodological quality of Case-control Studies according to the NEWCASTLE-OTTAWA Quality Assessment Scale

| **Study** | **Selection** | | | | **Comparability** | **Exposure** | | | **Total** |
| --- | --- | --- | --- | --- | --- | --- | --- | --- | --- |
|  | **Is the case definition adequate** | **Representativeness**  **of the cases** | **Selection of**  **controls** | **Definition of**  **controls** | **Comparability of cases and controls on the basis of the design or analysis** | **Ascertainment of exposure** | **Same method of ascertainment for cases and controls** | **Non-Response rate** |  |
| Voigt LF,  et al | ★ | ★ | ★ | ★ | ★ | ★ | ★ | / | **7 Stars** |
| Symmons DP, et al | ★ | / | ★ | ★ | ★ | / | ★ | / | **5 Stars** |
| Uhlig T,  et al | ★ | ★ | ★ | ★ | ★★ | / | ★ | / | **7 Stars** |
| Pedersen M, et al | ★ | ★ | ★ | ★ | ★★ | ★ | ★ | / | **8 Stars** |
| Rodríguez LA, et al | ★ | ★ | ★ | ★ | ★ | ★ | ★ | ★ | **8 Stars** |
| Wesley A,  et al | ★ | ★ | ★ | ★ | ★ | ★ | ★ | ★ | **8 Stars** |
| Crowson CS, et al | ★ | / | ★ | ★ | ★ | ★ | ★ | ★ | **7 Stars** |

Supplementary. Table: Methodological quality of Cohort Studies according to the NEWCASTLE-OTTAWA Quality Assessment Scale

| **Study** | **Selection** | | | | **Comparability** | **Outcome** | | | **Total** |
| --- | --- | --- | --- | --- | --- | --- | --- | --- | --- |
|  | **Represen**  **-tativeness of the exposed cohort** | **Selection of**  **the non exposed cohort** | **Ascertainment of exposure to implants** | **Demonstration that outcome of interest was not present at start of study** | **Comparability of cohorts on the basis of the design or analysis** | **Assessment of outcome** | **Was follow up long enough for outcomes to occur** | **Adequacy of follow up of cohorts** |  |
| Cerhan JR, et al | ★ | ★ | ★ | ★ | ★ | ★ | ★ | ★ | **8 Stars** |
| Lahiri M,  et al | ★ | ★ | ★ | ★ | ★ | ★ | ★ | ★ | **8 Stars** |
| Lu, et al | / | ★ | ★ | ★ | ★★ | ★ | ★ | ★ | **8 Stars** |
| Harpsøe MC, et al | ★ | ★ | ★ | ★ | ★ | ★ | ★ | ★ | **8 Stars** |
