# Supplementary figures and images for: Body mass index and the risk of rheumatoid arthritis: a systematic review and dose-response meta-analysis
Source: Arthritis Res Ther. 2015 Mar 29;17(1):86. doi: 10.1186/s13075-015-0601-x (PMC4422605; doi:10.1186/s13075-015-0601-x)

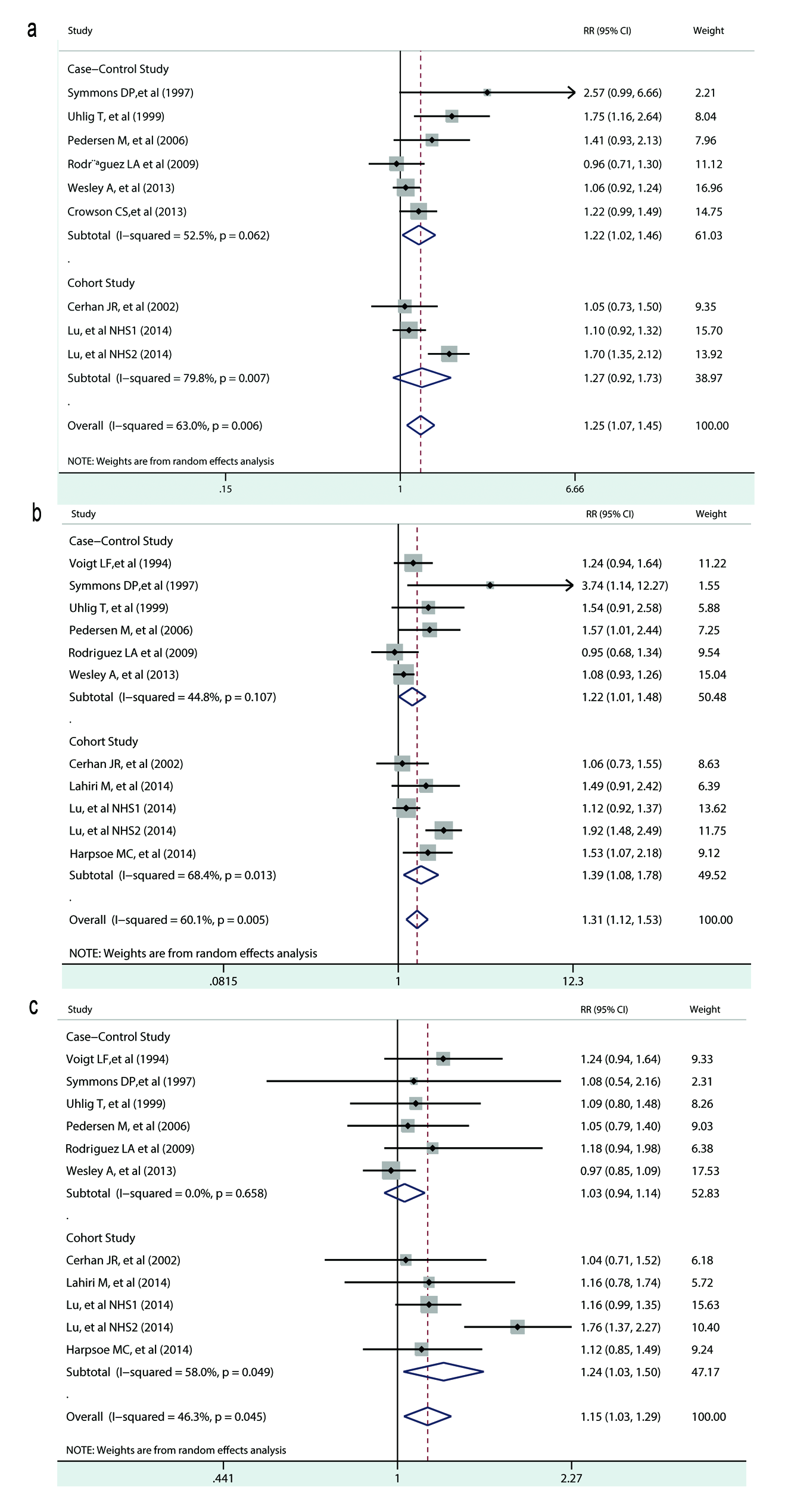

Supplement: Additional file 2: — Overall RR of rheumatoid arthritis stratified by study design. (a) obesity versus non-obesity; (b) obesity versus normal weight; (c) overweight versus normal weight; meta-analyses using a random-effects model. CI: confidence interval; RR: relative risk. [file 13075_2015_601_MOESM2_ESM.tiff]

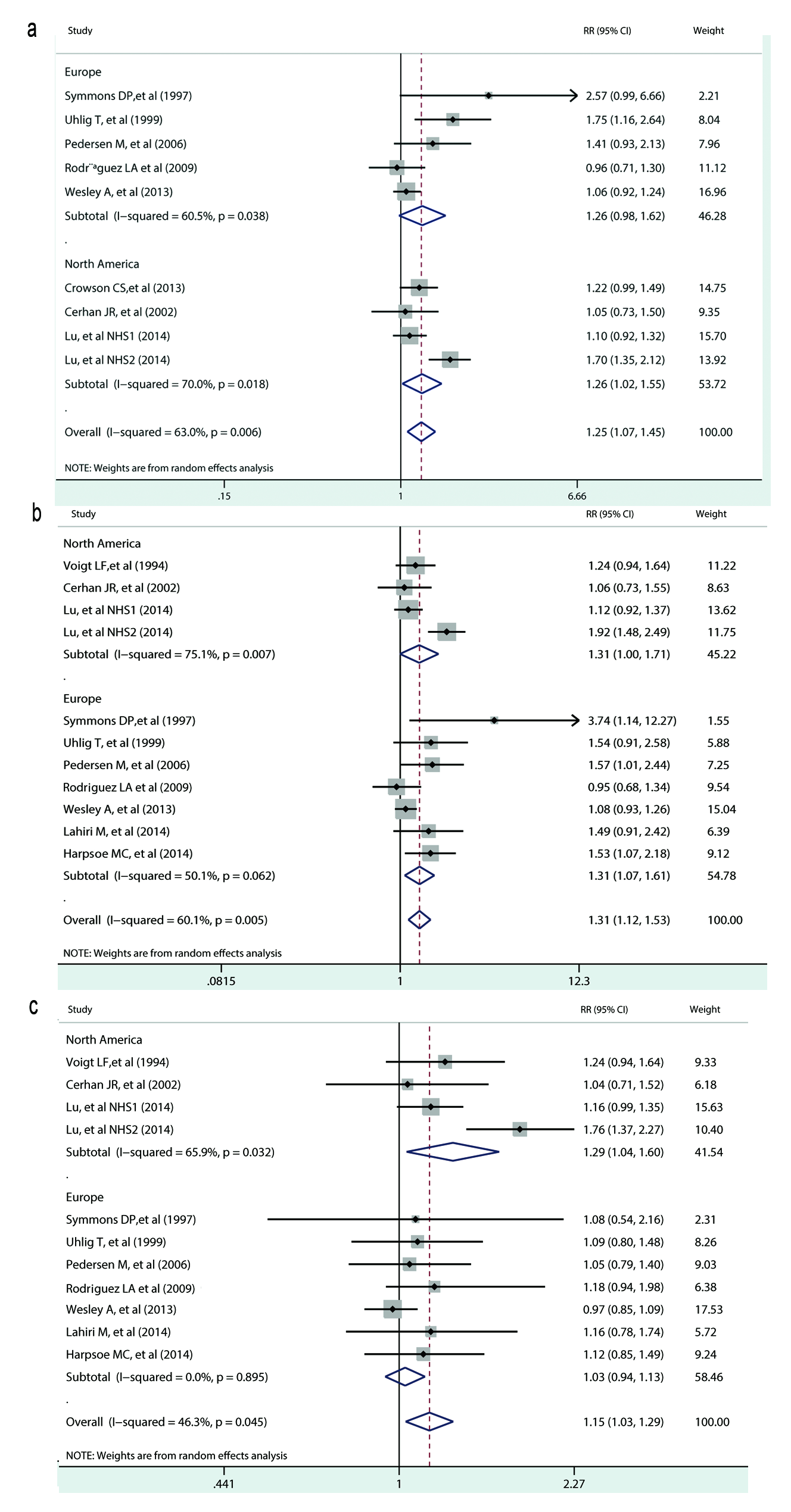

Supplement: Additional file 3: — Overall RR of rheumatoid arthritis stratified by region. (a) obesity versus non-obesity; (b) obesity versus normal weight; (c) overweight versus normal weight; meta-analyses using a random-effects model. CI: confidence interval; RR: relative risk. [file 13075_2015_601_MOESM3_ESM.tiff]

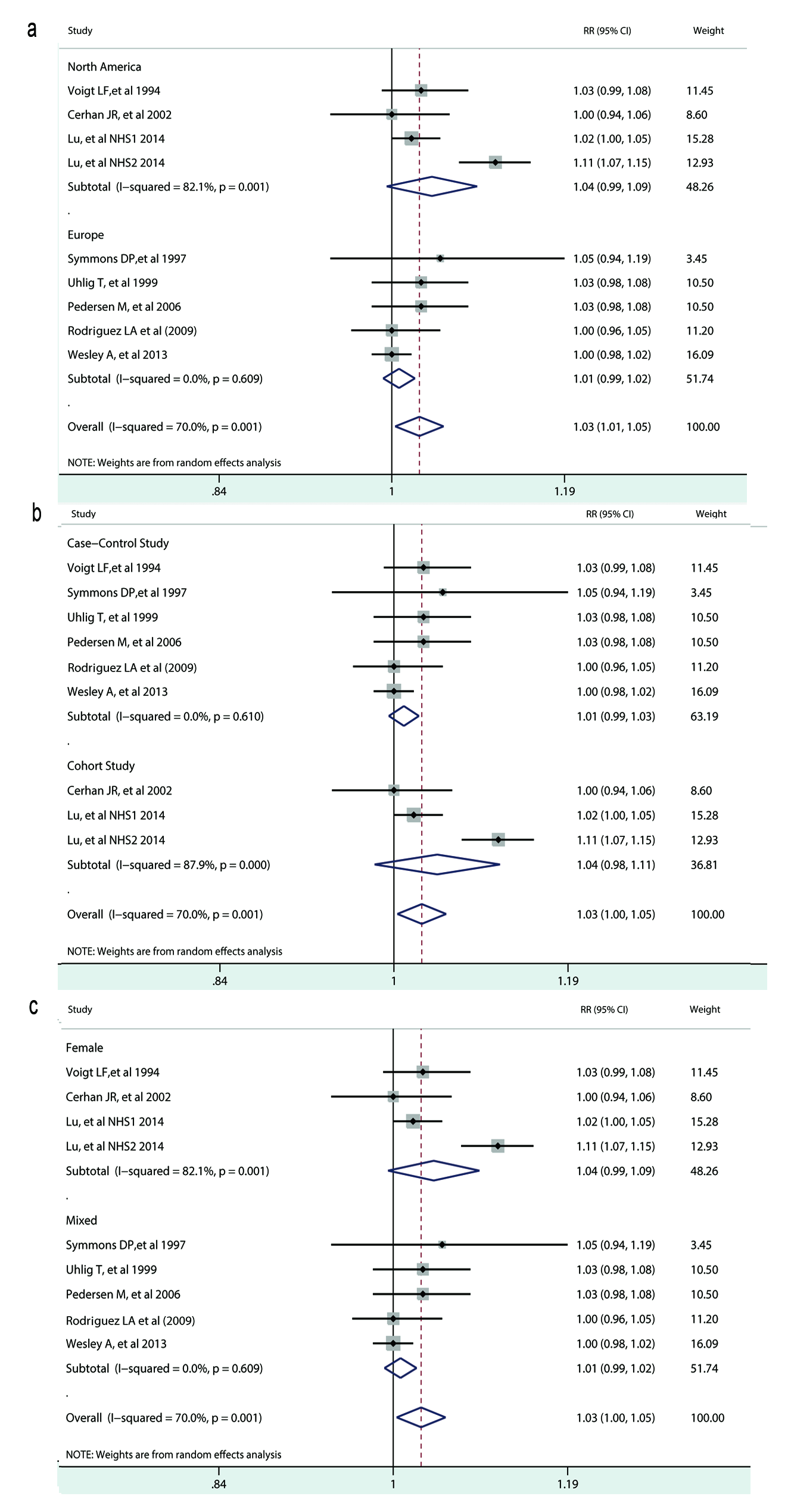

Supplement: Additional file 4: — RRs of rheumatoid arthritis risk per 5 kg/m2 increase in body mass index stratified by region, study design, and gender. (a) region; (b) study design; (c) gender; meta-analyses using a random-effects model. CI: confidence interval; RR: relative risk. [file 13075_2015_601_MOESM4_ESM.tiff]

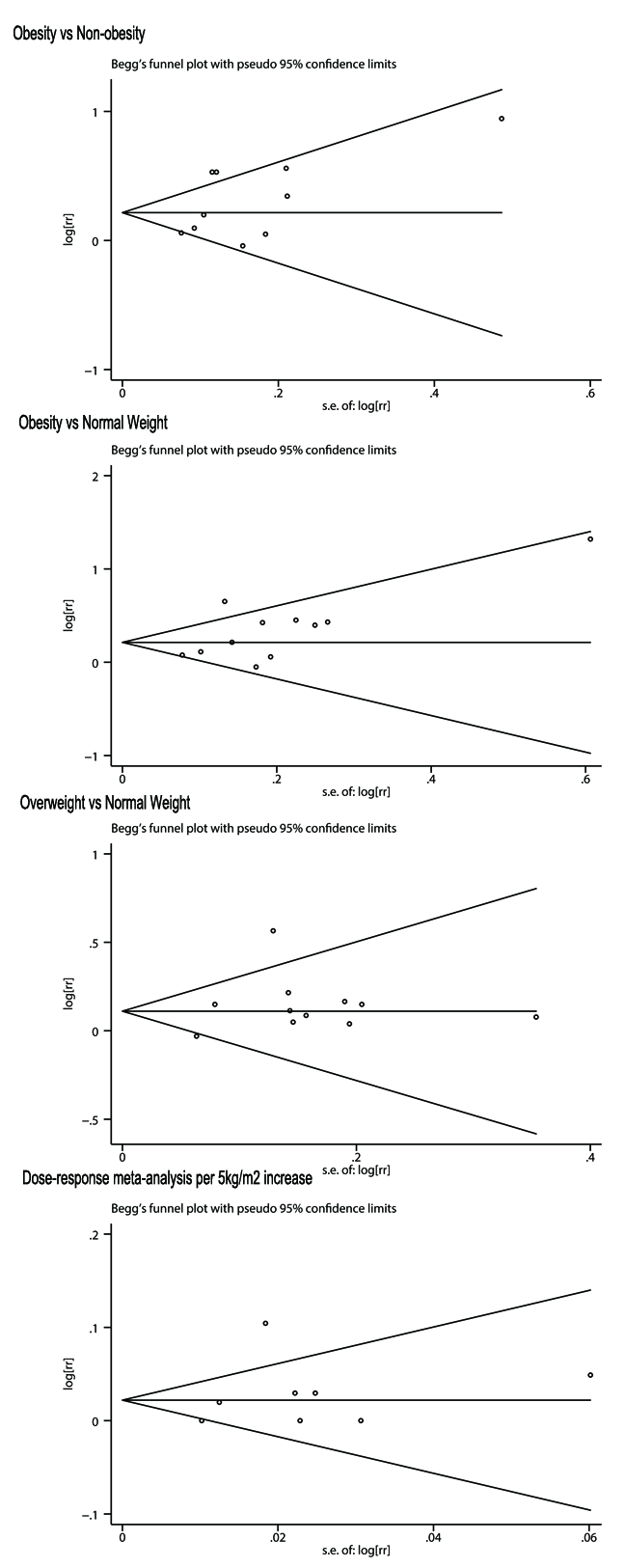

Supplement: Additional file 5: — Funnel plots for obesity versus non-obesity, obesity versus normal weight, overweight versus normal weight, and dose-response per 5 kg/m2 increase in body mass index. RR: relative risk. [file 13075_2015_601_MOESM5_ESM.tiff]

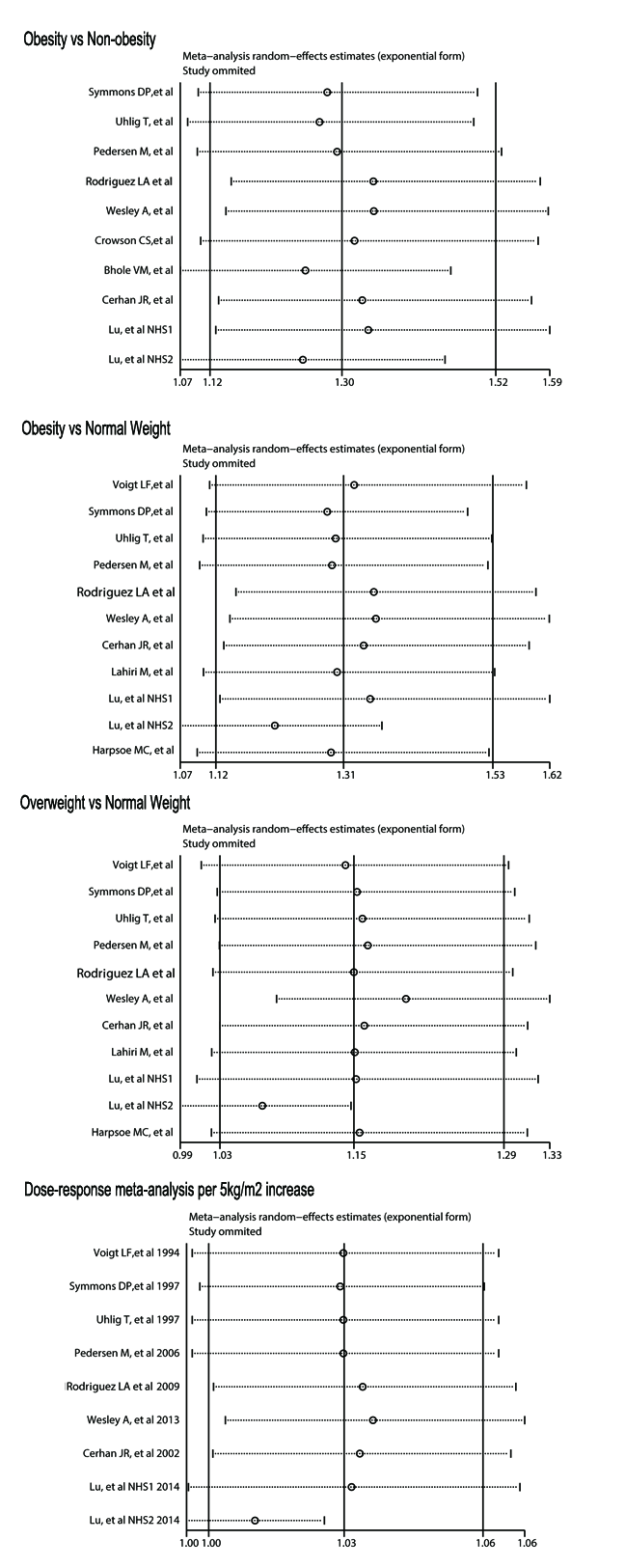

Supplement: Additional file 6: — Sensitivity analyses for obesity versus non-obesity, obesity versus normal weight, overweight versus normal weight, and dose-response by per 5 kg/m2 increase in body mass index. [file 13075_2015_601_MOESM6_ESM.tiff]
